# *Alpinia katsumadai* and *Wurfbainia vera* Extracts Modulate Antioxidant Function and Intestinal Morphology in Danzhou Chickens via Gut Microbiota–Metabolite Interactions Involving Hydroxyoctadecadienoic Acid Metabolism and *Bacteroidota* Remodeling

**DOI:** 10.3390/microorganisms14030703

**Published:** 2026-03-20

**Authors:** Hongzhi Wu, Haoliang Chai, Xilong Yu, Dexin Zhao, Hanyang Liu, Weiqi Peng, Fengjie Ji, Liangmei Xu, Guanyu Hou

**Affiliations:** 1 Tropical Crops Genetic Resources Research Institute, Chinese Academy of Tropical Agricultural Sciences, Haikou 571101, China; hong-zhi@163.com (H.W.); pwq0912@163.com (W.P.); fengjie_ji@126.com (F.J.); 2 College of Animal Science and Technology, Northeast Agricultural University, Harbin 150030, China; chaihaoliang@163.com (H.C.); yuxl0467@163.com (X.Y.); zhaodexin20020126@yeah.net (D.Z.); xuliangmei@neau.edu.cn (L.X.); 3 School of Clinical Medicine, Hainan Medical University, Haikou 571101, China; hyliu2025@163.com

**Keywords:** Danzhou chicken, *Alpinia katsumadai*, *Wurfbainia vera*, antioxidant performance, intestinal morphology

## Abstract

This study evaluated the effects of supplementing *Alpinia katsumadai* and *Wurfbainia vera* extracts on the growth performance, antioxidant capacity, intestinal metabolites, and microbiota of Danzhou chickens. Using Danzhou broilers, we examined the individual or combined inclusion of *Alpinia katsumadai* and *Wurfbainia vera* extracts in a 2 × 2 factorial layout. Four hundred and eighty female dual-purpose chickens were randomly assigned to four treatments (six replicates of 20 chicks each): control basal diet (CON), basal + 600 mg kg^−1^ *Alpinia katsumadai* (T1), basal + 600 mg kg^−1^ *Wurfbainia vera* (T2), or basal + 600 mg kg^−1^ *Alpinia katsumadai* + 600 mg kg^−1^ *Wurfbainia vera* (T3). All treatments differed significantly from CON. For intestinal morphology, T1, T2, and T3 increased jejunal villus height and villus-to-crypt ratio while reducing crypt depth. T1 exceeded CON (*p* < 0.05), and an interaction was detected. T1 raised the abundances of *Bacteroidota*, *Bifidobacterium*, *Tidjanibacter*, and *Phocaeicola* relative to CON (*p* < 0.05). T3 exhibited higher activities of glutathione peroxidase and catalase than CON, T2, and T1 (*p* < 0.05). Metabolomically, T1, T2, and T3 elevated intestinal Menaquinone-9, lecithin, and L-galactono-1,5-lactone versus CON (*p* < 0.05). T3 lowered 3-(R)-Hydroxyoctadecadienoic acid and 9-(R)-Hydroxyoctadecadienoic acid versus CON and T1, and increased eugenol versus CON (*p* < 0.05). Overall, T1 and T2, especially in combination, enhance antioxidant capacity, improve gut morphology, promote beneficial microbiota and activate health-related metabolic pathways in Danzhou broilers.

## 1. Introduction

Intestinal barrier dysfunction initiates gut microbial dysbiosis, whose disordered community directly compromises intestinal structure and function while also elevating systemic oxidative stress via microbial metabolites; this oxidative stress in turn further disrupts the intestinal milieu by inhibiting beneficial bacteria and promoting pathogenic bacteria, thereby establishing a self-amplifying, growth-suppressing, and economically costly vicious cycle [1]. In high-density poultry production systems, chickens are continuously exposed to stressors that disrupt gut microbial homeostasis, compromise intestinal barrier integrity, and initiate a vicious cycle of inflammation, malabsorption, and nutrient loss. Consequently, this culminates in reduced growth performance, impaired feed conversion efficiency, and heightened susceptibility to pathogens [2,3]. For decades, low-dose antibiotic growth promoters have been routinely administered in intensive poultry farming based on the premise that they confer intestinal health benefits and mitigate disease incidence. However, this strategy originally devised to optimize nutritional utilization has, via widespread overuse, fostered the accumulation of antibiotic resistance genes and persistent drug residues. The European Union, China, and the United States have fully banned it; although Brazil enacted a ban in 2003, most antibiotics are still authorized for use under regulation [4]. These contaminants now pervade the food chain, posing substantial threats to both food safety and public health [5]. Motivated by the global shift toward antibiotic-free animal production, the livestock sector and research community face the pressing need to develop natural alternatives that are both safe and effective. In this context, plant extracts—particularly those capable of modulating gut microbiota while exerting anti-inflammatory and antioxidant activities—represent a promising and readily applicable strategy for sustaining microbial homeostasis and supporting intestinal health [6,7].

Chinese medicinal herbs, by virtue of their natural origin, the minimal residue they leave, and their comparatively lower propensity to elicit microbial resistance, have come to be regarded as among the most promising candidates poised to replace antibiotics in an era increasingly constrained by drug-resistant pathogens. *Alpinia katsumadai* and *Wurfbainia vera*, two congeneric species within the Zingiberaceae that find their principal Chinese habitats in the humid tropical zones of Hainan, Guangdong, and Guangxi, have, by virtue of their simultaneous status as food and as medicine, long been enlisted both in the therapeutic repertoire of human disease and in the flavoring traditions of regional Chinese cuisine [8,9,10,11,12]. As traditionally used Zingiberaceous plants with both culinary and medicinal applications, *Alpinia katsumadai* and *Wurfbainia vera* possess a wide array of bioactive compounds such as flavonoids, diarylheptanoids, and essential oils; however, existing research remains fragmented in that studies on *Alpinia katsumadai* have largely concentrated on alpinetin, whereas investigations into *Wurfbainia vera* have predominantly focused on eucalyptol [13,14]. The fruits, roots, fibrous roots, stems, leaves, and shells of *Alpinia katsumadai* possess a diverse profile of volatile organic compounds, all of which show some degree of antimicrobial potency [15]. Research conducted in porcine [16] and murine [17] models has substantiated that extracts derived from *Alpinia katsumadai* exhibit a range of notable biological activities, such as antioxidant, anti-inflammatory, antimicrobial, and meat quality-enhancing properties. Antimicrobial assays demonstrated that eucalyptol, obtained by steam distillation of mature *Wurfbainia vera* fruits, inhibits the growth of *Escherichia coli*, *Staphylococcus aureus*, *Enterococcus faecalis*, *Streptococcus agalactiae*, and *Streptococcus dysgalactiae* [18]. Notably, *Alpinia katsumadai* and *Wurfbainia vera* both contain 1,8-cineole, a compound recognized for conferring antimicrobial and antioxidant effects in various Chinese herbal medicines such as *Atractylodes lancea* and *Agastache rugosa* [19]. Yuting et al. [20] demonstrated that when Ross 308 broilers received dietary supplementation of 1,8-cineole at 10–40 mg/kg, the birds exhibited an enhanced antioxidant capacity and strengthened immune responses, which collectively contributed to improved growth performance. In a murine model of depression, Wu et al. [21] demonstrated that 1,8-cineole activates the Nrf2/HO-1 signaling pathway, enhances superoxide dismutase activity, and diminishes malondialdehyde levels, thus establishing a mechanistic foundation for its antioxidant properties in chickens. Furthermore, 1,8-cineole induced a significant increase in ileal villus height and V/C in broilers [20], and moreover, it enhanced intestinal muscularis thickness in fish [22], with these structural improvements ultimately serving to optimize intestinal morphology and promote growth.

Danzhou chicken, a dual-purpose indigenous chicken breed in Hainan Province, demonstrates strong stress tolerance but is characterized by a slow growth rate, which restricts its economic and commercial potential [23,24,25,26]. *Alpinia katsumadai* and *Wurfbainia vera* are traditional medicinal herbs abundant in bioactive constituents such as 1,8-cineole. However, their potential application as feed additives in poultry nutrition, especially in indigenous chicken breeds, remains largely under-investigated [27,28]. Therefore, the present study was designed to investigate the individual and combined effects of dietary *Alpinia katsumadai* and *Wurfbainia vera* extracts on growth performance, antioxidant capacity, and intestinal health in Danzhou chickens. To elucidate the underlying mechanisms from a microbe–host co-metabolism perspective, we integrated intestinal microbiota profiling and host metabolomic analyses. We hypothesized that dietary supplementation with *Alpinia katsumadai* and *Wurfbainia vera* extracts could enhance growth performance and intestinal health in Danzhou chickens. Given their distinct yet complementary bioactive profiles, the combination of *Alpinia katsumadai* and *Wurfbainia vera* is expected to exert synergistic effects, resulting in greater improvements in antioxidant capacity and gut microbiota modulation compared with either extract alone. Through systematic evaluation of these parameters, this study aims to provide a theoretical foundation for developing novel, green feed additives derived from Hainan’s indigenous medicinal plants, thereby supporting the global initiative to reduce antibiotic usage in animal production.

## 2. Materials and Methods

### 2.1. Animal Ethics Statement

All management and procedures described in this article were approved by the Institutional Animal Care and Use Committee of the Chinese Academy of Tropical Agricultural Sciences (approval number: CATAS20250319-3). These studies were conducted in accordance with local legislation and institutional requirements. Written informed consent was obtained from the owners for the participation of their animals in this study.

### 2.2. Experiment Materials

The extracts of *Alpinia katsumadai* and *Wurfbainia vera* were sourced from Shaanxi Baichuan Biotechnology Co., Ltd. (Xi’an, China). The *Alpinia katsumadai* extract yielded 1.80% volatile oil, which consisted of 86.91% terpenoids and 3.74% flavonoids. The characteristic components were 1,8-cineole (43.70%) and cardamonin (1.35%). The *Wurfbainia vera* extract yielded 5.32% volatile oil, with 93.28% terpenoids. The major components were 1,8-cineole (82.40%) and a trace of amomum ketone (0.24%). These components were identified using high-performance liquid chromatography and mass spectrometry at the Chinese Academy of Tropical Agricultural Sciences (Haikou, China).

*Alpinia katsumadai* and *Wurfbainia vera* extracts were used in powdered form and the mixing process employed in this study was designed to ensure uniform distribution of all components in the feed. Specific procedures included first placing all conventional raw materials into a small-scale grinder (9FC21) for co-grinding to achieve the standard particle size in accordance with the nutritional specifications of the experimental basal diet. The ground materials were then accurately weighed and transferred to a drum mixer (Model: YGH 100; Shanghai Ni′er Machinery Manufacturing Co., Ltd., Shanghai, China) followed by the addition of precisely weighed powdered extracts of *Alpinia katsumadai* and *Wurfbainia vera*; the mixture was continuously blended at a rotational speed of 18 r/min for 45 min to ensure thorough and homogeneous dispersion of the extracts within the basal diet. Finally, the well-mixed powder was processed into pelleted feed using a pellet mill (Model: RT 2S; Henan Changxing Machinery Equipment Co., Ltd., Zhengzhou, China) for experimental applications.

One-day-old female dual-purpose Danzhou chickens, with an initial body weight of 24.42 ± 0.41 g, were procured from the Danzhou Chicken Breeding Base in Danzhou City, Hainan Province. These chickens shared uniform genetic backgrounds and health statuses.

### 2.3. Experiment Design and Management

This study utilized a 2 × 2 factorial design. A total of 480 chickens were randomly assigned to four groups, with six replicates per group and 20 chickens in each replicate. The control group (CON) received a basal diet, while the experimental groups were supplemented with 600 mg/kg of *Alpinia katsumadai* extract (T1), 600 mg/kg of *Wurfbainia vera* extract (T2), or a combination of both (T3, each at 600 mg/kg) in the basal diet. The dosage levels were determined based on the results of preliminary experiments. The basal diet for the chickens was formulated in accordance with the feeding standards outlined in the National Research Council (1994) guidelines, and the composition and nutritional levels of the basal diet are presented in Table 1. Before chick placement, the house was pre-heated to ensure floor temperature exceeded air temperature, and chicks were monitored for behavioral signs of thermal stress. The experimental period spanned 35 days. Throughout this period, the chicken house was maintained at a temperature range of 23–27 °C and a relative humidity of 50–60%. The experiment was conducted in a closed, windowless poultry house equipped with mechanical ventilation and an evaporative cooling system. LED lighting (cool white, 5000 K) provided a photoperiod of 16 h light and 8 h darkness per day, with light intensity maintained at 20 lux at bird level. The air exchange rate was adjusted according to the size of the chick flock and environmental conditions, with ventilation scheduled around 12 noon. During the experiment, Danzhou chickens were allowed to freely eat pellet feed and drink water. The feed was placed in concave plastic trays, and the remaining feed was weighed before each feeding. Drinking water was provided through nipple drinkers, and the water lines were cleaned once a week. Feeding times were fixed at 07:00 and 18:00 daily to ensure a consistent feeding pattern. The experiment employed a stocking density of 20 birds per square meter, with rice husks serving as the bedding material; the bedding was replaced every 5 days and maintained at a thickness of 10 cm.

### 2.4. Sample Collection

At the end of the trial, a 12 h fasting period was implemented, during which water was provided ad libitum. From each of the six replicate pens per treatment group, six female Danzhou chickens with body weights approximating the mean weight of their respective pen were chosen for blood collection, followed by slaughter and tissue sampling. The average daily feed intake (ADFI) was calculated by recording the total feed provided and the feed remnants in each pen daily, then dividing by the number of birds and the number of days. The average daily weight gain (ADG) was determined by measuring the body weight of each bird at the start and end of the trial and dividing the total weight gain by the number of days. The feed conversion ratio (FCR) was computed as the ratio of ADFI to ADG.

Prior to slaughter, blood was collected from the wing vein of the chickens using five mL vacuum blood collection tubes. The blood samples were allowed to clot at room temperature for 20 min, after which they were centrifuged at 3000× *g* for 20 min. The resulting serum was collected and stored at −20 °C for subsequent analysis. After slaughter, approximately 1 cm segments from the middle portions of the duodenum, jejunum, and ileum were excised. The intestinal contents were flushed with physiological saline, and the cleaned segments were fixed in 4% paraformaldehyde solution for histological analysis. Additionally, a segment from the middle portion of the cecum was excised, and the cecal contents were collected. These contents were transferred into cryovials and stored at −80 °C for further analysis.

### 2.5. Serum Biochemical Indicators

Serum samples were retrieved from a −20 °C freezer. The activities of glutathione peroxidase (GSH-Px), total superoxide dismutase (T-SOD), and catalase (CAT) were measured using a spectrophotometer (UV-2600, Shimadzu, Shanghai, China). The concentrations of interleukin-10 (IL-10), interleukin-6 (IL-6), and tumor necrosis factor-alpha (TNF-α) were determined using an enzyme-linked immunosorbent assay (ELISA) reader (Varioskan LUX, ThermoFisher, Shanghai, China). All assays were performed according to the protocols provided in the reagent kits (Nanjing Jiancheng Bioengineering Institute, Nanjing, China). All ELISA plates were incubated at 37 °C for 60 min, washed three times with PBS-T, and then incubated with substrate solution for 15 min at room temperature before stop solution was added. Absorbance was measured at 450 nm.

### 2.6. Intestinal Histological Morphology

The duodenum, jejunum, and ileum samples, after fixation, were processed for histological examination. These samples were dehydrated through a graded series of ethanol solutions, cleared in xylene, and embedded in paraffin wax. Sections of 4–5 µm thickness were cut using a microtome (Leica RM2255, Leica Microsystems, Wetzlar, Germany) and mounted on glass slides. The sections were then subjected to hematoxylin and eosin (H.E.) staining according to standard protocols. Briefly, the sections were dewaxed in xylene, rehydrated through graded alcohols, and stained with hematoxylin for nuclear staining, followed by eosin for cytoplasmic staining. After staining, the sections were dehydrated, cleared, and mounted with a coverslip using a mounting medium. Imaging of the stained sections was performed using a biological microscope (EVOS FL Auto, Thermo Fisher Scientific, Waltham, MA, USA). The villus height was measured from the tip of the villus to the base of the crypt, and the crypt depth was measured from the base of the crypt to the muscularis mucosae. These measurements were conducted using a pathological image analysis system (Motic Images Advanced 3.2, Motic China Group Co., Ltd., Xiamen, China). At least six well-oriented villi and crypts per section were measured, and the average values were calculated for each sample.

### 2.7. Cecal Content 16S rDNA Gene Sequencing

Genomic DNA extraction was performed using the E.Z.N.A.^®^ Soil DNA Kit (Omega Bio-tek, Norcross, GA, USA). The V3–V4 region of the bacterial 16S rRNA gene was amplified by PCR using barcoded specific primers 338F (5′-ACTCCTACGGAGGCAGCAG-3′) and 806R (5′-GGACTACHVGGGTWTCTAAT-3′). The amplification products were detected by 2% agarose gel electrophoresis, and target bands were excised and purified. The purified products were quantified using Qubit^®^ 4.0(Thermo Fisher Scientific, Waltham, MA, USA), and samples were pooled in equimolar amounts to construct an Illumina sequencing library. The specific steps included ligation of Y-shaped adapters, removal of self-ligated fragments through magnetic bead screening, and PCR enrichment of the library templates. The library was then denatured with sodium hydroxide to generate single-stranded DNA fragments. Sequencing was performed on the Illumina MiSeq PE300 (Illumina, Inc., San Diego, CA, USA), platform. Bridge PCR amplification was used to generate DNA clusters, and sequencing-by-synthesis (SBS) technology was employed, utilizing fluorescently labeled dNTPs, where fluorescence signals were captured to determine the sequence. Raw sequencing data were quality-controlled using fastp, and denoising and Amplicon Sequence Variant (ASV) feature table generation were performed using QIIME2 (version 2022.8). Specifically, the DADA2 (q2-dada2 version 2024.10.0) plugin within QIIME2 was employed for denoising, chimera removal, and ASV inference, using default parameters unless otherwise stated. Taxonomic annotation was performed using the RDP Classifier (confidence threshold 0.7) against the Silva 138.1 database. Intestinal content samples were submitted to the NovoMagic Cloud Platform at https://magic.novogene.com/ (accessed on 13 July 2025) for microbial 16S rDNA sequencing and analytical identification. All sequencing data from this study have been deposited in the NCBI Sequence Read Archive under the BioProject accession number PRJNA1348103.

### 2.8. Cecal Content Metabolomics Analysis

Liquid chromatography–tandem mass spectrometry (LC–MS; Waters Corporation, Milford, MA, USA) was employed for the analysis of metabolites. Chromatographic separation of metabolites was achieved using a Waters ACQUITY UPLC HSS T3 column (2.1 mm × 100 mm, 1.8 μm). During sample preparation, metabolites were extracted using methanol containing the internal standard 2-chloro-L-phenylalanine. The extraction procedure was conducted through a series of steps, including tissue homogenization, ultrasonication, centrifugation, and filtration, ultimately yielding the supernatant. The analysis was performed utilizing a Thermo Vanquish liquid chromatography system interfaced with a Q Exactive mass spectrometer (Thermo Fisher Scientific, Waltham, MA, USA). Data acquisition was executed in both positive and negative ionization modes. The extraction procedure was conducted through a series of steps, including tissue homogenization, ultrasonication, centrifugation, and filtration, ultimately yielding the supernatant. The analysis was performed utilizing a Thermo Vanquish liquid chromatography system interfaced with a Q Exactive mass spectrometer. Data acquisition was executed in both positive and negative ionization modes. Mass spectrometric parameters included an electrospray ionization source, full scan, and data-dependent MS/MS. The acquired data were subjected to preprocessing using Pareto-scaling, followed by multivariate statistical analysis in SIMCA-P software (version 14.1, Umetrics, Umeå, Sweden). This analysis included principal component analysis (PCA) and orthogonal partial least squares-discriminant analysis (OPLS-DA) to construct classification models. The reliability of these models was evaluated using permutation testing. Additionally, variable importance in projection (VIP) scores were calculated from the OPLS-DA model to identify the most influential variables in the classification.

### 2.9. Quantitative Real-Time PCR (qPCR)

Total RNA was extracted from 1.5–2.0 g of liver tissue using an animal tissue RNA extraction kit(RNeasy Mini Kit, QIAGEN GmbH, Hilden, Germany). RNA quality and integrity were verified by 2% agarose gel electrophoresis, while concentration and purity were assessed using a NanoPhotometer^®^ (Implen GmbH, Munich, Germany). Samples with an A260/A280 ratio between 1.8 and 2.1 were used for downstream analyses. Primer sequences are listed in Table 2. cDNA was synthesized using the PrimeScript^®^ RT Reagent Kit with gDNA Eraser (TaKaRa, Dalian, China). For genomic DNA removal, 2 μL of 5× gDNA Eraser Buffer, 1 μL of gDNA Eraser, and 1 μg of total RNA were combined and adjusted to a final volume of 10 μL with RNase-free water. Reverse transcription was then performed in a 20 μL reaction containing 10 μL of the gDNA-treated mixture, 1 μL of RT Primer Mix, 4 μL of 5× PrimeScript Buffer, 1 μL of PrimeScript RT Enzyme Mix I, and 4 μL of RNase-free water. The thermal program consisted of 37 °C for 15 min and 85 °C for 5 s, after which cDNA samples were stored at −20 °C until use.

Quantitative real-time PCR (qPCR) was carried out on an Applied Biosystems PRISM 7500 Fast Real-Time PCR System (Applied Biosystems, Foster City, CA, USA) using SYBR^®^ Premix Ex Taq II (TaKaRa). Each 20 μL reaction contained 10 ng of cDNA template and 0.4 μM of each forward and reverse primer (Sangon Biotech, Shanghai, China). The amplification protocol included an initial denaturation at 95 °C for 30 s, followed by 40 cycles of 95 °C for 5 s and 60 °C for 34 s. A melting curve analysis was subsequently performed from 60 °C to 95 °C, with 0.3 °C increments every 15 s. *Glyceraldehyde-3-phosphate dehydrogenase* (*GAPDH*) and *β-actin* (*ACTB*) were used as internal reference genes. *GAPDH* and *ACTB* were chosen as reference genes based on their well-documented stable expression in avian tissues and their widespread use in poultry nutritional and physiological studies. Their expression levels remained consistent across all experimental groups in our pilot tests, confirming their suitability for normalizing target gene expression in this model. The relative mRNA expression levels of *GPX1* (*Glutathione Peroxidase 1*), *SOD1* (*Superoxide Dismutase 1*), *EGF* (*Epidermal Growth Factor*), *PCNA* (*Proliferating Cell Nuclear Antigen*), *TRPV1* (*Transient Receptor Potential Cation Channel Subfamily V Member 1*), and *LRAT* (*Lecithin Retinol Acyltransferase*) were quantified using the 2^−ΔΔCt^ method and normalized to the average expression of the two reference genes.

### 2.10. Statistical Analysis

All data were statistically analyzed using SPSS 22.0 software (IBM SPSS Statistics, Chicago, IL, USA). Intergroup comparisons were performed using two-way analysis of variance (ANOVA), followed by post hoc multiple comparisons using the LSD test. Experimental data are presented as the mean ± standard deviation. A *p*-value of less than 0.05 was considered statistically significant, and different uppercase and lowercase letters were used in the figures to denote significant differences. Graphs were generated using GraphPad Prism 10.0 software (GraphPad Software Inc., San Diego, CA, USA), and illustrations were created using FigDraw (Home for Researchers, Hangzhou, China; www.figdraw.com) accessed on 15 January 2025. The following model was used:

Y_ij_ = μ + α_i_ + β_j_ + α_i_ × β_j_ + e_ij,_

Y_ij_ is the observed value under the i-th level of *Alpinia katsumadai* and the j-th level of *Wurfbainia vera*; α_i_ is the fixed effect of *Alpinia katsumadai* (i = 1, 2. representing absence or presence); β_j_ is the fixed effect of *Wurfbainia vera* (j = 1, 2. representing absence or presence); α_i_ × β_j_ is the interaction effect between *Alpinia katsumadai* and *Wurfbainia vera*; e_ij_ is the random residual. When the interaction was not significant, experimental groups were compared within the pooled data. If *Alpinia katsumadai* or *Wurfbainia vera* was significant, it was reported as a main effect.

Differential metabolites were identified based on the following three criteria:

Variable importance in projection (VIP) from the PLS-DA model > 1.0;

Absolute log_2_ fold change (|log_2_FC|) > 0.58;

Adjusted *p*-value (Bonferroni-corrected) < 0.05.

Prior to statistical analysis, metabolomics data were normalized and log-transformed to meet the assumptions of parametric tests.

## 3. Results

### 3.1. Effects on Production Performance in Danzhou Chickens

Compared with the CON group, the T1, T2, and T3 groups showed no significant differences (*p* > 0.05) in production performance, and no interaction (*p* > 0.05) was observed (Table 3).

### 3.2. Effects on Biochemical Indicators in Danzhou Chickens

The GSH-Px content in the T3 group was significantly higher than that in the CON, T1, and T2 groups (*p* < 0.05). Regarding CAT activity, the T1 group was significantly lower than the T3 group (*p* < 0.05), while the CAT content in the T3 group was significantly higher compared to both the CON and T2 groups (*p* < 0.05). Co-supplementation with *Alpinia katsumadai* and *Wurfbainia vera* extracts resulted in significant differences in GSH-Px and CAT levels compared to individual supplementation (*p* < 0.05), indicating an interactive effect. No significant differences were observed in any serum inflammatory cytokine indicators (*p* > 0.05), and no interaction (*p* > 0.05) was detected (Table 4).

### 3.3. Effects on Intestinal Histomorphology in Danzhou Chickens

**Figure 1:**
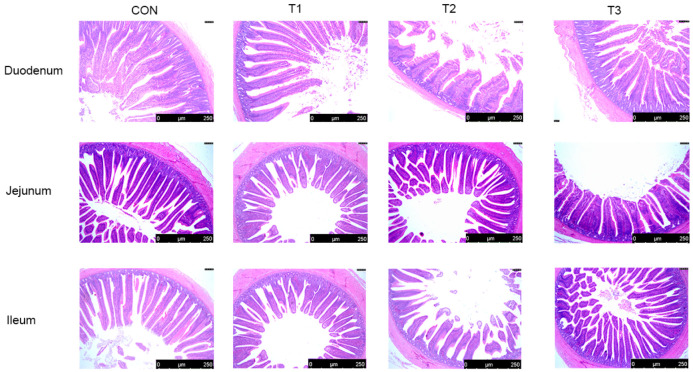
Effects of *Alpinia katsumadai* and *Wurfbainia vera* extracts on intestinal morphology in Danzhou chickens. CON is the control group; T1 is supplemented with *Alpinia katsumadai* extract; T2 is supplemented with *Wurfbainia vera* extract; and T3 is supplemented with a mixture of both *Alpinia katsumadai* and *Wurfbainia vera* extracts. *n* = 6 per group.

In the jejunum, all supplementation groups exhibited significantly reduced crypt depth compared to the CON group (*p* < 0.05). The villus height to crypt depth ratio (V/C) was significantly higher in the T1 group than in the CON group (*p* < 0.01), while the T2 and T3 groups also showed significantly elevated V/C ratios compared to the CON group (*p* < 0.05). Co-supplementation with *Alpinia katsumadai* and *Wurfbainia vera* extracts resulted in significant differences in jejunal villus height, crypt depth, and V/C ratio compared to individual supplementation, revealing a significant interaction (*p* < 0.05) (Table 5, Figure 1).

### 3.4. Effects on the Cecal Microbiota of Danzhou Chickens

#### 3.4.1. 16S rDNA Verification

**Figure 2:**
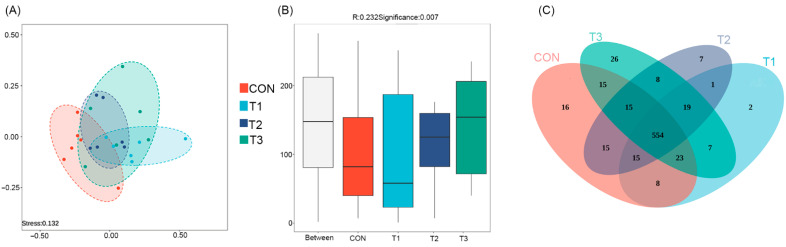
Analysis of differences in gut microbiota among groups and distribution of unique OTUs. (**A**) NMDS analysis; (**B**) anosim analysis; (**C**) Venn diagram analysis. CON is the control group; T1 is supplemented with *Alpinia katsumadai* extract; T2 is supplemented with *Wurfbainia vera* extract; and T3 is supplemented with a mixture of both *Alpinia katsumadai* and *Wurfbainia vera* extracts. *n* = 6 per group.

Non-metric multidimensional scaling (NMDS) revealed a stress value of 0.132 (a stress value < 0.2 indicates that the two-dimensional NMDS plot is meaningful and interpretable), while analysis of similarities (ANOSIM) yielded an R-value of 0.232 (Figure 2A,B). The results demonstrate a significant difference in beta diversity between the CON group and the supplemented groups. Venn diagram analysis indicated that the T3 group had the most pronounced effect on the gut microbiota structure, exhibiting 26 unique operational taxonomic units (OTUs). The CON, T2, and T1 groups contained 16, 7, and 2 unique OTUs, respectively (Figure 2C).

#### 3.4.2. Changes in the Diversity of the Cecal Microbiota

**Figure 3:**
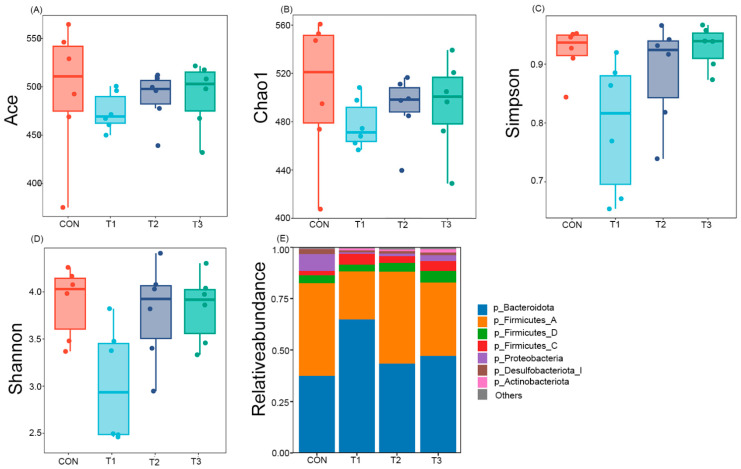
Analysis of alpha diversity and species composition of gut microbiota in each group. (**A**) Ace Exponential box plot; (**B**) Chao1 Exponential box plot; (**C**) Simpson Exponential box plot; (**D**) Shannon Exponential box plot; (**E**) analysis of bar map of phylum community. CON is the control group; T1 is supplemented with *Alpinia katsumadai* extract; T2 is supplemented with *Wurfbainia vera* extract; and T3 is supplemented with a mixture of both *Alpinia katsumadai* and *Wurfbainia vera* extracts. *n* = 6 per group.

In the alpha diversity analysis, the Ace and Chao1 indices were used to assess species richness in the gut, while the Simpson and Shannon indices were employed to evaluate species diversity. The results revealed no significant differences (*p* > 0.05) among the groups in the cecal microbial communities of Danzhou chickens, indicating comparable levels of microbial species richness and evenness across groups (Figure 3A–D). Further investigation into the relative abundance of gut microbiota at different taxonomic levels demonstrated that supplementation with *Alpinia katsumadai* and *Wurfbainia vera* extracts modulated the composition of the gut microbiota (Figure 3E). At the phylum level, the gut microbiota of Danzhou chickens was predominantly composed of five phyla: *Bacteroidota, Firmicutes, Proteobacteria, Desulfobacterota_I*, and *Actinobacteria*.

#### 3.4.3. LEfSe Analysis of Cecal Microbiota

**Figure 4:**
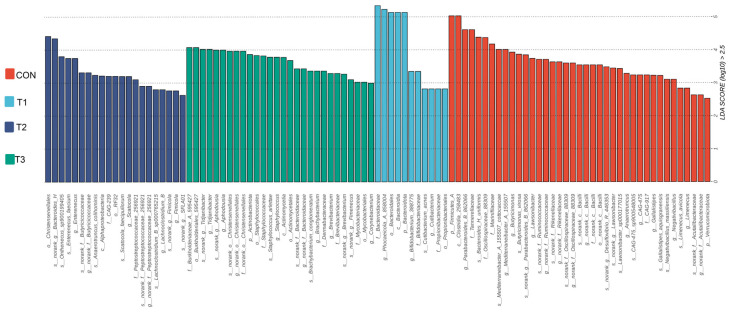
Identification Lda effect size analysis bar chart. CON is the control group; T1 is supplemented with *Alpinia katsumadai* extract; T2 is supplemented with *Wurfbainia vera* extract; and T3 is supplemented with a mixture of both *Alpinia katsumadai* and *Wurfbainia vera* extracts. *n* = 6 per group.

Employing LEfSe analysis, we identified differentially abundant bacterial taxa across various treatment groups, detecting a total of 99 significantly enriched taxonomic clades from phylum to species level (Figure 4). At the phylum level, the relative abundance of *Bacteroidota* was significantly higher in the T1 group compared to the CON group (*p* < 0.01). On the family level, the relative abundance of *Bacteroidaceae* was markedly elevated (*p* < 0.01) in the T1 group relative to the CON group. Furthermore, at the genus level, the abundance of *Tidjanibacter* was substantially higher (*p* < 0.01) in both the T3 and T1 groups than in the CON group, with the T3 group exhibiting the highest levels. Additionally, the abundance of *Bifidobacterium* and *Phocaeicola* was significantly greater (*p* < 0.05) in the T1 group compared to both the CON and T3 groups. Detailed differences in microbial abundance across groups are presented in Figure S1.

### 3.5. Effects on the Cecal Content Metabolism of Danzhou Chickens

#### 3.5.1. Quality Assessment of Metabolomics Data

**Figure 5:**
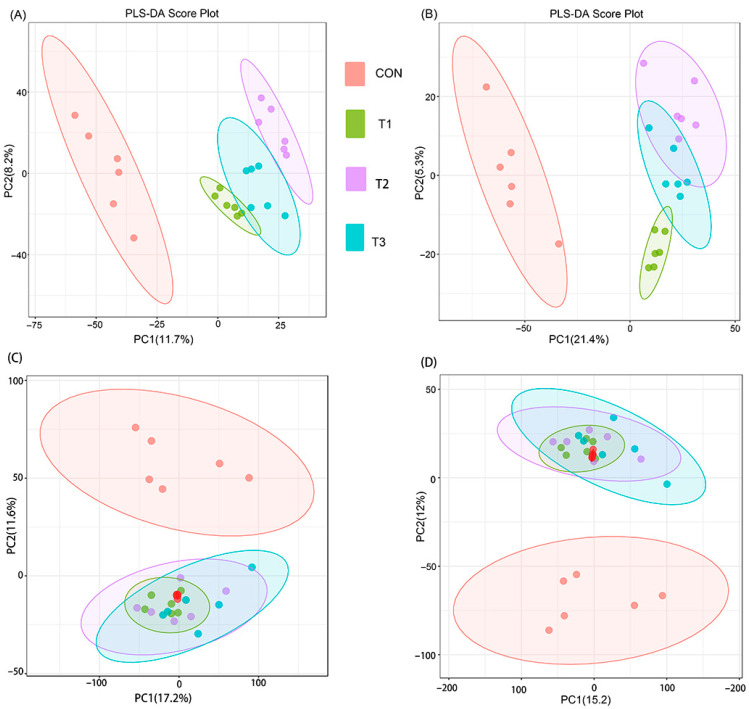
Multivariate statistical analysis of gut metabolites in each group. (**A**) PLS-DA in each group of positive ion modes; (**B**) PLS-DA in each group of negative ion modes. (**C**,**D**) The PCA score plot of QC samples in both positive and negative ion modes shows red dots representing QC samples and other colored dots representing experimental samples. The QC samples exhibit a clustering trend within the 95% confidence interval, demonstrating satisfactory reproducibility. CON is the control group; T1 is supplemented with *Alpinia katsumadai* extract; T2 is supplemented with *Wurfbainia vera* extract; and T3 is supplemented with a mixture of both *Alpinia katsumadai* and *Wurfbainia vera* extracts. *n* = 6 per group.

To investigate the regulatory effects of *Alpinia katsumadai* and *Wurfbainia vera* on the intestinal metabolic profile of Danzhou chickens, untargeted metabolomics was performed. PLS-DA score plots in both positive and negative ionization modes revealed distinct and separate clustering of intestinal metabolites between the CON group and the T1, T2, and T3 groups (Figure 5A,B). Permutation testing results under both positive and negative ionization modes confirmed the reliability of the PLS-DA models without overfitting (Figure S2). PCA score plots in both ionization modes demonstrated good reproducibility and correlation, indicating clear differences in metabolites between the T1, T2, and T3 groups compared to the CON group (Figure 5C,D).

#### 3.5.2. Screening of Metabolites and Metabolic Pathways

**Figure 6:**
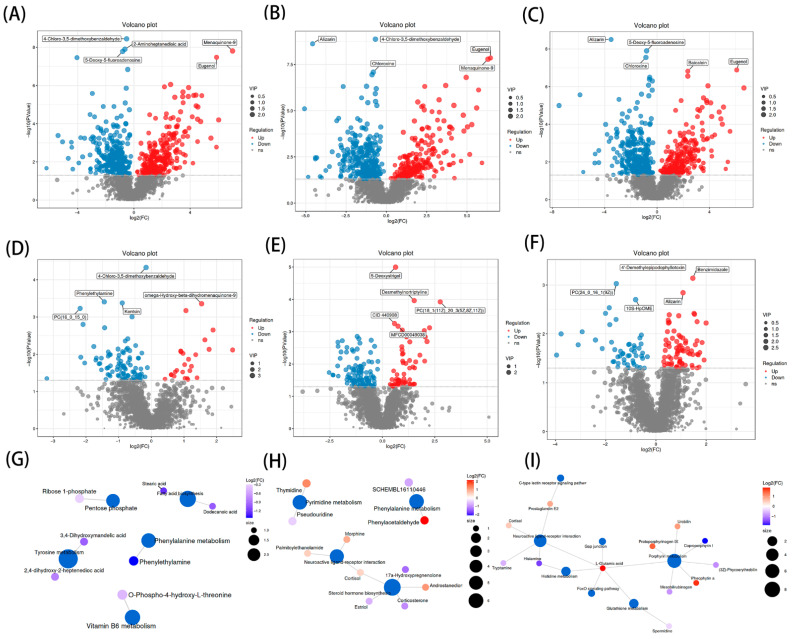
Volcano plots and KEGG enrichment network of differential metabolites. (**A**) T1 vs. CON Volcanic difference map; (**B**) T2 vs. CON Volcanic difference map; (**C**) T3 vs. CON Volcanic difference map; (**D**) T2 vs. T1 Volcanic difference map; (**E**) T3 vs. T1 Volcanic difference map; (**F**) T3 vs. T2 Volcanic difference map; (**G**) T2 vs. T1 KEGG Enrichment analysis network diagram; (**H**) T3 vs. T1 KEGG Enrichment analysis network diagram; (**I**) T3 vs. T2 KEGG Enrichment analysis network diagram. CON is the control group; T1 is supplemented with *Alpinia katsumadai* extract; T2 is supplemented with *Wurfbainia vera* extract; and T3 is supplemented with a mixture of both *Alpinia katsumadai* and *Wurfbainia vera* extracts. *n* = 6 per group.

The overall metabolite clustering heatmap revealed distinct clustering between the CON group and the supplemented groups, indicating clear intergroup differences in metabolites (Figure S3). Differential volcano plots highlighted significantly altered metabolites, demonstrating that compared to the CON group, the T1, T2, and T3 groups exhibited prominent upregulation of Menaquinone-9 and eugenol (Figure 6A–F). Based on the Z-score plot of differential metabolites and the KEGG enrichment analysis bubble chart, the primary differential metabolic pathways identified among CON vs. T1, T2, and T3 were mainly enriched in Linoleic acid metabolism and ascorbate and aldarate metabolism. Key metabolites associated with these two pathways included lecithin, 3-HODE, 9-HODE, and L-galactono-1,5-lactone, as illustrated in the mechanistic diagram (Figure S4). To better compare the CON, T1, T2, and T3 groups, further KEGG enrichment analysis was performed on the differential metabolites. The results, interpreted through a metabolic pathway impact factor network diagram incorporating Impact value, Hits, and *p*-value, are shown (Figure 6G–I). Changes in the content of differential metabolites and pathway-related metabolites across groups are presented in Figure S5.

#### 3.5.3. Combined Analysis of 16S rDNA and Metabolomics

**Figure 7:**
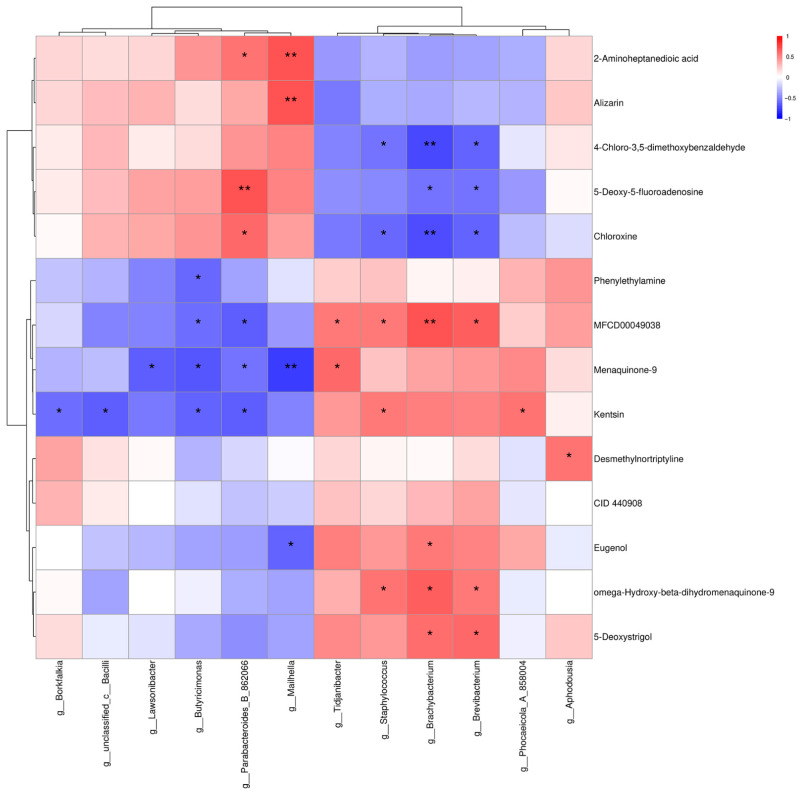
Spearman correlation analysis of significant gut microbiota and metabolite parameters in Danzhou chickens supplemented with *Alpinia katsumadai* and *Wurfbainia vera* extracts. The horizontal axis represents microbes, and the vertical axis represents metabolites. Red indicates positive correlations, blue indicates negative correlations; * denotes significance (*p* < 0.05), ** denotes high significance (*p* < 0.01). *n* = 6 per group.

To investigate the potential mechanisms by which *Alpinia katsumadai* and *Wurfbainia vera* improve the growth performance of Danzhou chickens, Spearman correlations between gut microbiota and intestinal metabolites were further analyzed. As shown in Figure 7, *g_Lawsonibacter* demonstrated a significant negative correlation with Menaquinone-9. *G_Butyricimonas* was significantly negatively correlated with Menaquinone-9 (*p* < 0.05). *g_Mailhella* showed a significant negative correlation with Eugenol (*p* < 0.05) and a highly significant negative correlation with Menaquinone-9 (*p* < 0.01). *Tidjanibacter* was significantly positively correlated with Menaquinone-9 (*p* < 0.05). Chloroxine is significantly negatively correlated with *g_Staphylococcus*, *g_Brachybacterium*, and *g_Brevibacterium* (*p* < 0.05). However, 5-Deoxystrigol is significantly positively correlated with *g_Brachybacterium* and *g_Brevibacterium*.

#### 3.5.4. Analysis of Gene Expression Related to Intestinal Metabolites and Oxidative Stress Levels

**Figure 8:**
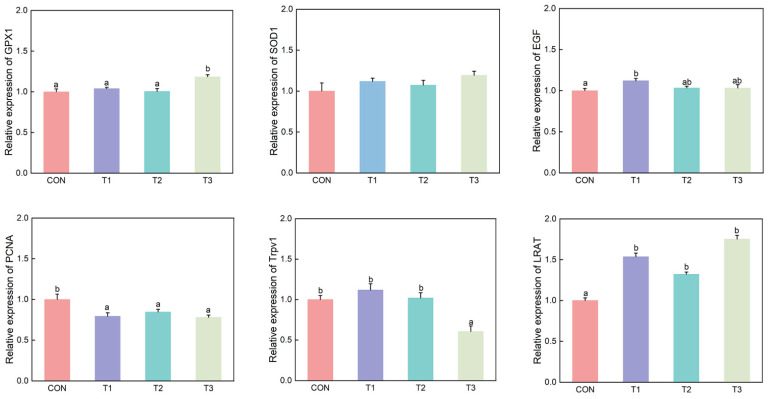
Gene expression in intestinal metabolites and oxidative stress. Values in the same column with different lowercase superscript letters denote significant differences (*p* < 0.05). CON is the control group; T1 is supplemented with *Alpinia katsumadai* extract; T2 is supplemented with *Wurfbainia vera* extract; and T3 is supplemented with a mixture of both *Alpinia katsumadai* and *Wurfbainia vera* extracts. *n* = 6 per group.

As shown in Figure 8, the expression levels of GPX1 and LRAT in the T3 group were significantly higher than those in the CON group, while Trpv1 was significantly lower than in the CON, T1, and T2 groups (*p* < 0.05). The levels of EGF and LRAT in the T1 group were significantly higher than those in the CON group, while the level of PCNA was significantly lower than that in the CON group (*p* < 0.05). LRAT levels in the T3 group were significantly higher than in the CON group, while PCNA levels were significantly lower than in the CON group (*p* < 0.05).

## 4. Discussion

The intestinal tract of the chicken constitutes an intricately orchestrated and functionally polyvalent ecosystem whose homeostatic condition operates as the pivotal determinant of both the efficiency with which dietary nutrients are assimilated and the magnitude of the bird’s overall productive output [29]. Within this system, the intestinal villi, which constitute a dense array of finger-like epithelial projections that carpet the luminal surface of the gut wall, function as the fundamental anatomical and physiological units responsible for the efficient absorption of nutrients, and their morphometric configuration is such that villus height serves as a direct and quantifiable determinant of the mucosal absorptive surface area, because any increment in height produces a proportional expansion of the epithelial interface available for transepithelial transport, which in turn translates into an enhanced digestive and absorptive capacity [30]. Crypts, which are positioned at the base of the villous structures, progressively deepen in response to mucosal injury induced by stress, toxins, or pathogenic invasion, while the loss of epithelial cells initiates a marked acceleration in crypt cell proliferation as a reparative mechanism to restore epithelial continuity [31]. A higher V/C ratio is widely regarded as an indicator of enhanced intestinal health and greater absorptive potential [32]. The present investigation demonstrates that dietary supplementation with *Alpinia katsumadai* extracts as a single additive elicited a pronounced amelioration of ileal morphological architecture in Danzhou chickens, manifesting as a significant increment in villus height concomitant with a significant diminution in crypt depth, whereas neither the solitary administration of *Wurfbainia vera* nor the combined provision of both botanical extracts induced comparable morphometric changes. Although direct empirical scrutiny of *Alpinia katsumadai* incorporated into poultry diets for the purpose of modulating intestinal morphology remains absent from the extant literature, the seed’s signature flavonoid, cardamonin, has been documented to exert demonstrable anti-inflammatory and antioxidant bioactivities that plausibly underpin the observed histological improvements [33]. In investigations conducted by Wu et al. [34] cardamonin was demonstrated to elicit a marked improvement in the intestinal morphological parameters of weaned piglets, as evidenced by a significant increase in villus height, a corresponding reduction in crypt depth, and a consequent elevation in the villus height-to-crypt depth ratio. Findings that are closely aligned with the enhanced intestinal morphology and polymerase chain reaction (PCR) observations reported in the present study. Furthermore, 1,8-cineole, which occurs as a predominant constituent in both *Alpinia katsumadai* and *Wurfbainia vera*, has been demonstrated to elicit a pronounced amelioration of intestinal villus architecture and to exert a robust trophic effect on mucosal development when incorporated as a dietary supplement in chickens [35], and fish [36]. Improvements in intestinal morphology did not translate into enhanced production performance. This seemingly contradictory finding indicates that optimization of intestinal morphology does not always exert an immediate or direct effect on overall production performance. Multiple factors may underlie this observation. First, improved intestinal morphology can be considered an indicator of enhanced potential for nutrient absorption yet the realization of this potential is modulated by the body’s overall metabolic balance, prioritization of energy allocation and growth stage with a potential time-lag in its conversion to production performance [37]. Second, healthy animals may possess intrinsic physiological compensatory mechanisms through which nutrients or energy conserved via elevated absorption efficiency are reallocated to other homeostatic processes including immune surveillance and intestinal barrier maintenance thus failing to induce significant differences in growth-related parameters [38]. It is important to acknowledge that while the observed improvements in villus height and crypt depth are classically associated with enhanced absorptive surface area and reduced mucosal turnover, this study did not directly measure nutrient digestibility or absorption efficiency. Therefore, the inference that these morphological changes translate into enhanced functional nutrient utilization remains based on established physiological principles rather than direct empirical evidence from this experiment. Future studies incorporating digestibility trials or using markers of nutrient transport (e.g., gene expression of nutrient transporters) would be valuable to confirm the functional consequences of the observed morphological improvements.

The beneficial effects of *Alpinia katsumadai* extract on intestinal morphology are closely linked to its ability to modulate the gut microbial community and optimize its composition. *Bacteroidota*, a taxonomically coherent and metabolically versatile phylum that consistently ranks among the most abundant constituents of the avian intestinal microbiome, comprises a quantitatively conspicuous fraction of the microbial assemblage inhabiting the gastrointestinal tract of chickens whose homeostatic status is regarded as clinically normal [39]. This phylum possesses the metabolic capacity to ferment otherwise indigestible dietary fiber into a spectrum of SCFAs that exert pleiotropic beneficial effects on the host [40], and these microbially derived SCFAs subsequently stimulate the proliferation of intestinal epithelial cells, reinforce the structural and functional integrity of the epithelial barrier by providing a preferred energy substrate for colonocytes, and thereby create a physiological environment conducive to the elongation and maturation of the intestinal villi [41].

The present study demonstrated that, among all the experimental cohorts, the T1 group was distinguished by the most pronounced augmentation in the relative abundance of the phylum *Bacteroidota* together with the genera *Bifidobacterium* and *Phocaeicola*. Jiang et al. [42] reported that dietary supplementation with cardamonin increased the proportion of *Bacteroidota* in the gut of Danzhou chickens. Concurrently, research by Baiheng et al. [39] showed that supplementing the diet of Lohmann Brown laying hens with *Atractylodes lancea* and *Agastache rugosa*, which share active components with *Alpinia katsumadai*, significantly improved ileal crypt depth and *Bacteroidota* abundance, and altered the diversity and composition of the gut microbiota, which aligns with the findings of this experiment. *Phocaeicola* has been identified as a dominant degrader in the cecum of 35-day-old broilers, primarily breaking down mucin and complex polysaccharides, thereby contributing to microbial community maturation and diversity [43]. Furthermore, McKay et al. [44] in a mouse model, found that *Phocaeicola* alleviated ethanol- or high-fat-induced villus tip exfoliation and cryptitis by reducing lipopolysaccharide production and increasing butyrate levels, indirectly preserving villus integrity. Additionally, research by Liangyu et al. [32] in chicks revealed that *Bifidobacterium* in jejunal content and mucosa upregulates the expression of *Wnt/β-catenin* and proliferating cell nuclear antigen. The marked acceleration of Lgr5^+^ intestinal stem cell proliferation within the crypt base drives a pronounced upward flux of nascent epithelial progeny along the villus axis, a kinetic shift that culminates in a measurable increment in villus height concomitant with a reciprocal reduction in crypt depth and therefore engenders a significant elevation of the villus-to-crypt ratio [45,46]. These findings are consistent with the results of this study, where supplementation with *Alpinia katsumadai* extract increased the abundance of *Bifidobacterium* and *Phocaeicola*, and was associated with increased jejunal villus height, decreased crypt depth, and an elevated villus-to-crypt ratio. In summary, dietary supplementation with *Alpinia katsumadai* extracts in Danzhou chickens directly improves intestinal morphology through its active components, optimizes the gut microbiota structure by increasing beneficial bacteria such as *Bacteroidota*, *Bifidobacterium*, and *Phocaeicola*, and collectively supports gut health while alleviating dysbiosis.

Oxidative stress is a condition in which the generation of reactive oxygen species, arising from metabolic processes and environmental challenges, exceeds the body’s antioxidant defense capacity, thereby causing damage to cellular macromolecules [47]. This imbalance can consequently impair growth performance and compromise overall health in animals. Lymphokines, including IL-4, IL-6, and TNF-α, together with hepatic antioxidant enzymes such as SOD, GSH-Px, and CAT, constitute two interrelated and indispensable indicator systems that provide a comprehensive evaluation of immune regulatory balance and redox homeostasis; collectively, these parameters serve as objective biochemical markers that accurately mirror the organism’s internal physiological condition and its level of oxidative or metabolic stress [48,49,50]. Considering the 2 × 2 factorial design of this study, we focused not only on the effects of adding *Alpinia katsumadai* and *Wurfbainia vera* individually, but also placed greater emphasis on analyzing the interaction effect when both were added together. The results of this study demonstrate that the combined supplementation of *Alpinia katsumadai* and *Wurfbainia vera* markedly enhanced the activities of key antioxidant enzymes, including GSH-Px and CAT, thereby indicating a synergistic effect in strengthening the antioxidant defense system. The potent antioxidant activities of 1,8-cineole have been demonstrated across multiple species, including chickens [20], rats [51], mice [52], and fish [53], indicating its broadly conserved biological functions. It enhances the body’s antioxidant defenses by elevating the activities of SOD and GSH-Px as well as total antioxidant status [20]. These mechanisms are consistent with the antioxidant effects observed in the present study, where combined supplementation of *Alpinia katsumadai* and *Wurfbainia vera* extracts significantly increased GSH-Px and CAT activities. It is important to note, however, that while previous studies have also reported anti-inflammatory effects of 1,8-cineole via suppression of the NF-κB pathway and reduction in pro-inflammatory cytokines, the present study did not detect significant differences in serum levels of IL-6, IL-10, or TNF-α in any of the supplemented groups compared to the control. This suggests that, under the non-challenged conditions of this experiment, the primary beneficial effect of these extracts was mediated through enhancement of antioxidant capacity rather than modulation of systemic inflammatory responses. The lack of inflammatory cytokine changes may be attributable to the low basal activation level of inflammatory pathways in healthy chickens, which limited the opportunity for further suppression.

The aforementioned phenomena were further elucidated at the metabolomic level, revealing the mechanistic basis for the observed synergistic interaction. The interactive effects of combined additions are particularly evident. The concurrent supplementation of *Alpinia katsumadai* and *Wurfbainia vera* extracts strengthens the body’s intrinsic antioxidant defense mechanisms by modulating targeted metabolic pathways responsible for maintaining redox homeostasis. 3-HODE and 9-HODE, which are products of linoleic acid oxidation, function as sensitive biomarkers of lipid peroxidation, and a reduction in their levels reflects an improved antioxidant defense system or a mitigation of oxidative stress [54]. 3-HODE and 9-HODE are produced through the hydrolysis of lecithin catalyzed by phospholipase A2 and lecithin–cholesterol acyltransferase, resulting in the release of free linoleic acid, which is then subjected to oxidation by 12/15-lipoxygenase or through non-enzymatic processes under conditions of oxidative stress [55]. The concurrent increase in 3-HODE, 9-HODE, and lecithin within the intestine has been consistently observed across humans [56], mice [57], rats [58], and plants [59], representing a definitive indicator of activation of the linoleic acid metabolic pathway. This study demonstrated that the CON, T1, and T2 groups exhibited elevated levels of 3-HODE, 9-HODE, and lecithin, corroborating the findings reported in previous studies. In contrast, the T3 group displayed a distinct metabolic profile, characterized by a higher lecithin content concomitant with a reduction in 3-HODE and 9-HODE relative to the CON group. This decoupling of substrate and product indicates that the combined supplementation synergistically inhibits the downstream oxidation of linoleic acid or enhances the clearance of these peroxidation products, an effect not achieved by either supplement alone. Furthermore, the group receiving the combined supplementation presented with increased levels of L-galactono-1,5-lactone, a direct precursor in the ascorbate and aldarate metabolism pathway [60]. Catalyzed by L-galactono-1,5-lactone dehydrogenase, L-galactono-1,5-lactone undergoes direct conversion into ascorbic acid, thereby promoting vitamin C biosynthesis and consequently reinforcing the overall antioxidant defense system of the organism [61]. This further supports a synergistic interaction, where the combination uniquely unlocks the ascorbate and aldarate metabolism pathway, contributing to the bolstered antioxidant defense system observed via GSH-Px and CAT. Through the analysis of oxidative markers and gut metabolites, we arrived at this hypothesis. The combined supplementation of *Alpinia katsumadai* and *Wurfbainia vera* extracts exerted a synergistic effect in Danzhou chickens. 1,8-Cineole, a common constituent of both plants with potent antioxidant activity, likely plays a key role in this synergy. This combination markedly enhanced the activities of GSH-Px and CAT by modulating linoleic acid metabolism and ascorbate and aldarate metabolism pathways, promoting the accumulation of the key antioxidant precursor L-galactono-1,5-lactone, and simultaneously suppressing the formation of lipid peroxidation products 3-HODE and 9-HODE, thereby reinforcing the overall antioxidant defense capacity of the organism.

Eugenol has been shown to exert anti-inflammatory effects through a variety of mechanisms, encompassing the inhibition of bacterial proliferation, modulation of inflammatory cytokine expression, suppression of the NF-κB signaling pathway, and regulation of macrophage function, while simultaneously exhibiting potent antioxidant activity [62,63,64]. In this study, the intestinal content of Eugenol was significantly elevated in the T1, T2, and T3 groups relative to the CON group, with the highest concentration observed in the T3 group. Zhang et al. [65] reported that dietary supplementation with Eugenol in 1-day-old Arbor Acres plus male broilers markedly increased serum activities of GSH-Px, SOD, and T-AOC at 28 and 35 days of age. Complementary evidence supporting the antioxidant potential of Eugenol was provided by Alrashedi et al. [66] in meat rabbits, reinforcing its efficacy across species. These results are consistent with the elevated GSH-Px activity observed in the T3 group of our study, which exhibited the highest intestinal Eugenol concentration. Although eugenol has been reported to possess anti-inflammatory properties in other studies, the absence of significant changes in serum inflammatory cytokines in the present study indicates that the observed benefits were primarily antioxidant in nature under our experimental conditions. Vitamin K represents a critical dietary nutrient in intensive poultry production, with Menaquinone-9, a specific form of vitamin K_2_, playing a central role in the regulation of both blood coagulation and calcium metabolism [67,68,69], Our experimental findings suggest that the elevated intestinal concentrations of Menaquinone-9 observed in the supplemented groups may reflect either an enhancement in the absorption and metabolic utilization of dietary vitamin K or an upregulation of the capacity of specific gut microbiota to synthesize vitamin K_2_. Poultry are incapable of endogenous synthesis of vitamin K_2_, whereas members of the family *Bacteroidaceae* residing in their intestinal tract possess the capacity to biosynthesize Menaquinone-9 [70,71]. In the present study, the abundance of *Bacteroidaceae* was significantly higher in the T1, T2, and T3 groups than in the CON group, a finding that aligns with the report by Ozaki et al. [72] who observed a marked increase in intestinal *Bacteroidaceae* in animals receiving a high vitamin K_2_ diet compared with those receiving a low vitamin K_2_ diet. To explore potential associations between gut microbiota and vitamin K_2_ metabolism, we performed Spearman correlation analysis. The results revealed a significant positive correlation between Menaquinone-9 levels and the abundance of *Tidjanibacter*, a genus within the family *Rikenellaceae* (phylum *Bacteroidota*) [73]. This correlation suggests a potential link between this bacterial genus and vitamin K_2_ synthesis, although further studies are needed to establish causality. It is important to acknowledge that the correlation analyses performed in this study, while revealing significant associations between specific microbial taxa and metabolites, do not establish directionality or causality. The observed relationships could be bidirectional, or they might be influenced by confounding factors not measured in this study. Therefore, these findings should be interpreted with caution, and further mechanistic studies are necessary to confirm the functional roles of specific bacteria.

In the present experiment, no statistically significant differences were observed among treatments for growth performance parameters, including ADG, ADFI, FCR, or FBW. This apparent disconnect between the observed improvements in intestinal morphology, antioxidant capacity, and metabolite profiles and the lack of corresponding growth response raises important biological considerations. The absence of a growth effect may be attributed, at least in part, to the basal diet and husbandry conditions, which provided a healthy, non-challenged environment. Under such conditions, the growth potential of Danzhou chickens may have been fully realized, thereby limiting the scope for further improvement. The physiological enhancements induced by the *Alpinia katsumadai* and *Wurfbainia vera* extracts—such as strengthened antioxidant defenses and improved gut structure—are likely to function as stress-mitigating or prophylactic mechanisms, which may only translate into growth benefits under conditions of pathogen challenge or physiological stress. In the absence of such stressors, these improvements may not result in measurable gains in growth performance, possibly due to a partitioning of energy and nutrients toward the maintenance of heightened immune competence rather than toward growth-oriented processes. Furthermore, although the observed increases in villus height and reductions in crypt depth suggest enhanced intestinal absorptive capacity, it is possible that a longer duration of supplementation is required for these structural adaptations to cumulatively influence body weight gain. Another consideration relates to statistical power; while the current study was adequately powered to detect relatively large effects on sensitive physiological biomarkers, it may have been insufficient to detect smaller, yet biologically meaningful, differences in growth performance traits, which are typically characterized by higher variability. Collectively, these findings indicate that while the extracts exhibit clear potential to modulate gut health and systemic physiology, future studies incorporating challenge models (e.g., pathogen exposure or heat stress) or extending the supplementation period may be necessary to fully elucidate their growth-promoting potential under conditions.

## 5. Conclusions

**Figure 9:**
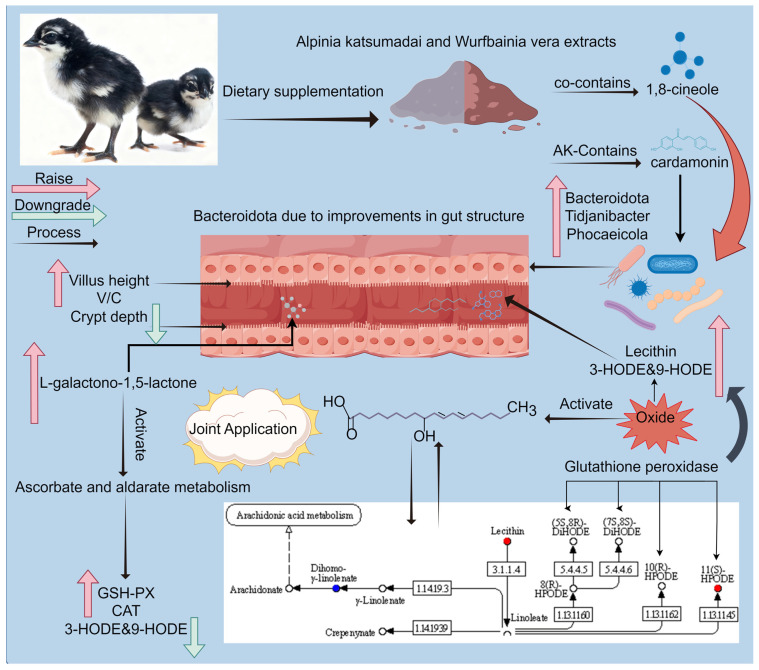
Mechanism of action diagram of *Alpinia katsumadai* and *Wurfbainia vera* extracts on Danzhou chickens. Adding *Alpinia katsumadai* and *Wurfbainia vera* to the diet of Danzhou chickens can improve intestinal morphology by increasing beneficial gut bacteria such as *Bacteroidota*, *Tidjanibacter*, and *Phocaeicola* through the action of their common component 1,8-cineole and cardamonin present in *Alpinia katsumadai*. Additionally, *Alpinia katsumadai* and *Wurfbainia vera* can alleviate oxidative stress by reducing the linoleic acid oxidation products 3-HODE and 9-HODE. 1.14.19.3, acyl-CoA 6-desaturase; 3.1.1.4, phospholipase A2; 5.4.4.5, 9,12-octadecadienoate 8-hydroperoxide 8R-isomerase; 5.4.4.6, 9,12-octadecadienoate 8-hydroperoxide 8S-isomerase; 1.14.19.39, Delta-9 fatty acid elongase; 1.13.11.60, 9S-lipoxygenase; 1.13.11.62, linoleate 10R-lipoxygenase; 1.13.11.45, linoleate 11-lipoxygenase; GSH-Px, glutathione peroxidase; CAT, catalase; V/C, Villus height/Crypt depth.

This study systematically investigated the effects of dietary supplementation with individual extracts of *Alpinia katsumadai* and *Wurfbainia vera*, as well as their combined administration, on Danzhou chickens. The results indicate that all supplemented groups showed measurable improvements relative to the control. Supplementation with *Alpinia katsumadai* alone was associated with the most pronounced improvements in intestinal morphology, including increased jejunal villus height, elevated villus-to-crypt ratio, reduced crypt depth, and higher abundances of *Bacteroidota*, *Bifidobacterium*, and *Phocaeicola*. Combined administration of *Alpinia katsumadai* and *Wurfbainia vera* extracts was associated with significantly enhanced serum antioxidant defenses, as reflected by elevated activities of glutathione peroxidase and catalase (Figure 9). Metabolomic analyses revealed associations between this combination and increased intestinal concentrations of eugenol and Menaquinone-9, reduced 3-HODE and 9-HODE levels, as well as modulation of the linoleic acid metabolic pathway. Based on these findings, dietary supplementation with 600 mg/kg of *Alpinia katsumadai* and 600 mg/kg of *Wurfbainia vera* may offer physiological benefits for Danzhou chickens, suggesting its potential as a natural feed additive to support gut health and antioxidant status.

Although no growth promotion was observed under stress-free conditions in this study, the improvements in intestinal and antioxidant indicators suggest that this combination could serve as a preventive strategy to enhance stress resistance in commercial production. Further studies employing mechanistic models are warranted to establish causal relationships and confirm the functional roles of specific microbial taxa and metabolites in mediating these effects. It is currently unclear whether lower doses of *Alpinia katsumadai* and *Wurfbainia vera* are equally effective, or whether higher doses can produce greater benefits. Future studies should test a range of concentrations (e.g., 300, 600, 900 mg/kg) to determine the most effective and economically feasible supplementation level.

## Figures and Tables

**Table 1:** Composition of the basal diet for Danzhou chickens, air-dried basis (%, kg/100 kg diet).

Ingredients	Contents	Nutrient Components	Contents
Corn	60.50	Metabolic energy ^2^, MJ/kg	13.65
Soybean meal	31.50	Crude protein ^3^	19.30
Soybean oil	1.30	Calcium ^3^	0.80
Calcium hydrogen phosphate	1.10	Available phosphorus ^3^	0.42
Salt	0.25	Lysine ^2^	0.93
Methionine	0.08	Methionine ^2^	0.45
Lysine (98%)	0.08	Cystine ^2^	0.35
Threonine	0.02	Threonine ^2^	0.73
Stone powder	0.90		
Fish meal	2.90		
Bran	0.37		
Premix ^1^	1.00		
Total	100		

^1^ The premix provides per kilogram of diet: VA 5000 IU, VD_3_ 3300 IU, VE 62.5 mg, VK 3.6 mg, VB_1_ 3.0 mg, VB_2_ 9.0 mg, VB_6_ 6.0 mg, VB_12_ 0.03 mg, folic acid 60 mg, niacin 60 mg, pantothenic acid 18 mg, biotin 0.36 mg, choline chloride 600 mg, Se as Na_2_SeO_3_ 0.33 mg, I as KIO_3_ 0.35 mg, Cu as CuSO_4_·5H_2_O 12 mg, Mn as MnSO_4_·H_2_O 60 mg, Fe as FeSO_4_·7H_2_O 80 mg, Zn as ZnSO_4_·7H_2_O 75 mg. ^2^ Calculated values. ^3^ Analyzed values.

**Table 2:** Primers used for quantitative RT-qPCR.

Genes	Primer Sequence (5′-3′)	Length of Product (bp)	Accession Number
*GPX1*	F:AACTGCAGGACGACATCGAG	182	NM_001277853
	R:CTTTGAAAACATCGGGCGCA		
*SOD1*	F:TGATGACCTGGGTAGAGGG	104	NM_205064
	R:ACAACGGTTAGCACTTGGCT		
*EGF*	F:AAATTTGTGGCGAGAGCGTC	177	NM_001001292
	R:TCGGCGTGTCTGACTACAAC		
*PCNA*	F:GACAATGCGGATACGTTGGC	188	NM_204170
	R:TCACCAATGTGGCTGAGGTC		
*Trpv1*	F:GAGTATGCCCAGAGCCCATC	93	NM_001001445
	R:CAGGCTGCTGTGTGGTAAGA		
*LRAT*	F:GATTTTGCCTATGGCGGCAG	197	XM_420371
	R:TTGTCGGTCTGGAAGCTGAC		
*ACTB*	F:GTGGATCAGCAAGCAGGAGT	182	NM_205518
	R:ATCCTGAGTCAAGCGCCAAA		
*GAPDH*	F:GTCAAGGCTGAGAACGGGAA	86	NM_204305
	R:GCCCATTTGATGTTGCTGGG		

*GPX1*, *Glutathione Peroxidase 1*; *SOD1*, *Superoxide Dismutase 1*; *EGF*, *Epidermal Growth Factor*; *PCNA*, *Proliferating Cell Nuclear Antigen*; *Trpv1*, *Transient Receptor Potential Cation Channel*, *Subfamily V*, *Member 1*; *LRAT*, *Lecithin Retinol Acyltransferase*; *ACTB*, *Actin*, *Beta*; *GAPDH*, *Glyceraldehyde-3-phosphate Dehydrogenase*.

**Table 3:** Growth performance of Danzhou chickens fed diets supplemented with *Alpinia katsumadai* and/or *Wurfbainia vera* extracts from 1 to 35 days of age.

Groups	Factors		IBW (g)	FBW (g)	ADG (g)	ADFI (g)	FCR
	Alpinia	Wurfbainia					
Con	0 mg/kg	0 mg/kg	23.57	257	6.67	14.75	2.21
T1	600 mg/kg	0 mg/kg	24.19	270	7.02	15.39	2.16
T2	0 mg/kg	600 mg/kg	23.31	265	6.91	15.41	2.30
T3	600 mg/kg	600 mg/kg	23.42	263	6.85	15.56	2.33
SEM			0.276	2.304	0.065	0.172	0.033
*p*-value							
T1	600 mg/kg	0 mg/kg	0.531	0.282	0.315	0.162	0.958
T2	0 mg/kg	600 mg/kg	0.386	0.928	0.838	0.143	0.059
T3	600 mg/kg	600 mg/kg	0.667	0.124	0.134	0.364	0.549
CV, %			5.250	3.900	4.221	4.993	6.667

Con: basal diet; T1: basal diet + 600 mg/kg *Alpinia katsumadai*; T2: basal diet + 600 mg/kg *Wurfbainia vera*; T3: basal diet + 600 mg/kg *Alpinia katsumadai* + 600 mg/kg *Wurfbainia vera*; SEM: standard error of the mean; CV, %: coefficient of variation, % (standard deviation/average) × 100%. IBW, initial body weight; FBW, final body weight; ADG, average daily gain; ADFI, average daily feed intake; FCR, feed conversion ratio. The sample consists of 4 groups with 6 replicates each, totaling 24.

**Table 4:** Serum antioxidant parameters of Danzhou chickens fed diets supplemented with *Alpinia katsumadai* and/or *Wurfbainia vera* extracts from 1 to 35 days of age.

Groups	Factors		GSH-Px, U/mL	SOD, U/mL	CAT, U/mL	IL-10, pg/mL	IL-6, pg/mL	TNF-α, pg/mL
	Alpinia	Wurfbainia						
Con	0 mg/kg	0 mg/kg	7927 ^a^	33.07	6.12 ^b^	277	49.99	1266
T1	600 mg/kg	0 mg/kg	8095 ^a^	33.36	4.65 ^ab^	231	35.60	1083
T2	0 mg/kg	600 mg/kg	7808 ^a^	29.01	2.71 ^aA^	241	56.51	1033
T3	600 mg/kg	600 mg/kg	9344 ^b^	37.70	8.86 ^cB^	309	28.53	809
SEM			205	1.325	0.670	14.474	6.061	102
*p*-value								
T1	600 mg/kg	0 mg/kg	0.013	0.082	0.009	0.689	0.101	0.351
T2	0 mg/kg	600 mg/kg	0.076	0.954	0.610	0.465	0.982	0.250
T3	600 mg/kg	600 mg/kg	0.037	0.102	0.001	0.057	0.580	0.924
CV, %			9.899	15.925	27.943	21.864	56.840	39.210

Con: basal diet; T1: basal diet + 600 mg/kg *Alpinia katsumadai*; T2: basal diet + 600 mg/kg *Wurfbainia vera*; T3: basal diet + 600 mg/kg *Alpinia katsumadai* + 600 mg/kg *Wurfbainia vera*; SEM: standard error of the mean; CV, %: coefficient of variation, % (standard deviation/average) × 100%. GSH-Px, glutathione peroxidase; SOD, total superoxide dismutase; CAT, catalase; IL-10, interleukin-10; IL-6, interleukin-6; TNF-α, tumor necrosis factor-alpha. ^a,b,c,A,B^ Within the same column, values with different lowercase letter superscripts denote significant differences (*p* < 0.05), while different uppercase letter superscripts indicate highly significant differences (*p* < 0.01). The same superscript letters or absence of superscript letters denote no significant difference (*p* > 0.05). The sample consists of 4 groups with 6 replicates each, totaling 24.

**Table 5:** Intestinal histomorphology of Danzhou chickens fed diets supplemented with *Alpinia katsumadai* and/or *Wurfbainia vera* extracts from 1 to 35 days of age (μm).

Groups	Factors		Duodenum			Jejunum			Ileum		
	Alpinia	Wurfbainia	Villus Height	Crypt Depth	V/C	Villus Height	Crypt Depth	V/C	Villus Height	Crypt Depth	V/C
Con	0 mg/kg	0 mg/kg	1309	251	5.93	800 ^a^	140 ^b^	5.77 ^aA^	791	147	5.79
T1	600 mg/kg	0 mg/kg	1326	161	8.47	897 ^b^	111 ^a^	8.17 ^B^	856	113	8.37
T2	0 mg/kg	600 mg/kg	1145	168	6.80	826 ^ab^	115 ^a^	7.24 ^b^	759	112	6.76
T3	600 mg/kg	600 mg/kg	1263	155	8.55	824 ^ab^	109 ^a^	7.61 ^b^	799	114	7.13
SEM			40.365	13.338	0.451	13.186	3.594	0.261	12.984	7.453	0.405
*p*-value											
T1	600 mg/kg	0 mg/kg	0.413	0.036	0.018	0.055	0.004	0.002	0.036	0.287	0.067
T2	0 mg/kg	600 mg/kg	0.175	0.062	0.570	0.325	0.020	0.257	0.069	0.259	0.860
T3	600 mg/kg	600 mg/kg	0.537	0.106	0.637	0.044	0.043	0.018	0.597	0.233	0.161
CV, %			15.623	35.487	29.698	7.722	14.814	17.775	7.934	29.826	28.276

Con: Dieta basal; T1: Dieta basal + 600 mg/kg *Alpinia katsumadai*; T2: Dieta basal + 600 mg/kg *Wurfbainia vera*; T3: Dieta basal + 600 mg/kg *Alpinia katsumadai* + 600 mg/kg *Wurfbainia vera*; SEM: standard error of the mean; CV, %: Coefficient variation, % (Standard deviation/Average) × 100%; V/C Villus height/Crypt depth. ^a,b,A,B^ Within the same column, values with different lowercase letter superscripts denote significant differences (*p* < 0.05), while different uppercase letter superscripts indicate highly significant differences (*p* < 0.01). The same superscript letters or absence of superscript letters denote no significant difference (*p* > 0.05). V/C, Villus height/Crypt depth. The sample consists of 4 groups with 6 replicates each, totaling 24.

## Data Availability

Sequence files associated with each sample have been submitted to the NCBI Sequence Read Archive (SRA accession number: PRJNA1348103; Public).

## Supplementary Materials

The following supporting information can be downloaded at: https://www.mdpi.com/article/10.3390/microorganisms14030703/s1, Gut Microbiota Statistical Test Bar Chart (Figure S1); Test results of arrangement under positive and negative ion modes (Figure S2); Differential metabolite overall analysis (Figure S3); Comparative analysis of metabolites and metabolic pathways among different additive groups (Figure S4); Statistical Test Bar Chart of Intestinal Metabolites (Figure S5). Table S1: Composition of the basal diet for Danzhou chickens, air-dried basis (% kg/100 kg diet)

## Abbreviations

The following abbreviations are used in this manuscript:

T1	*Alpinia katsumadai* extract group
T2	*Wurfbainia vera* extract group
T3	*Alpinia katsumadai* and *Wurfbainia vera* mixed extract group
3-HODE	3-Hydroxyoctadecadienoic Acid
9-HODE	9-Hydroxyoctadecadienoic Acid
VH/CD	Ratio of villus height to crypt depth
GSH-Px	Glutathione peroxidase
ADFI	Average daily feed intake
ADG	Average daily weight gain
FCR	Feed conversion Ratio
IL-10	Interleukin-10
IL-6	Interleukin-6
T-SOD	Total superoxide dismutase
TNF-α	Tumor necrosis factor-alpha
CAT	Catalase
*GPX1*	*Glutathione Peroxidase 1*
*SOD1*	*Superoxide Dismutase 1*
*EGF*	*Epidermal Growth Factor*
*PCNA*	*Proliferating Cell Nuclear Antigen*
*Trpv1*	*Transient Receptor Potential Cation Channel*
*LRAT*	*Lecithin Retinol Acyltransferase*
*ACTB*	*Actin*, *Beta*
*GAPDH*	*Glyceraldehyde-3-phosphate Dehydrogenase*
