# Supplementary material for: Alpinia katsumadai and Wurfbainia vera Extracts Modulate Antioxidant Function and Intestinal Morphology in Danzhou Chickens via Gut Microbiota–Metabolite Interactions Involving Hydroxyoctadecadienoic Acid Metabolism and Bacteroidota Remodeling
Source: Microorganisms. 2026 Mar 20;14(3):703. doi: 10.3390/microorganisms14030703 (PMC13029458; doi:10.3390/microorganisms14030703)
Supplement: Supplementary file 1 [file microorganisms-14-00703-s001.zip › microorganisms-4159997-supplementary.pdf]

## Supplementary materials

**Table S1.** Composition of the basal diet for Danzhou chickens, air-dried basis (% kg/100 kg diet)

| Ingredients | Contents | Nutrient components | Contents |
|---|---|---|---|
| Corn | 60.50 | Metabolic energy[2], MJ/kg | 13.65 |
| Soybean meal | 31.50 | Crude protein[3] | 19.30 |
| Soybean oil | 1.30 | Calcium[3] | 0.80 |
| Calcium hydrogen phosphate | 1.10 | Available phosphorus[3] | 0.42 |
| Salt | 0.25 | Lysine[2] | 0.93 |
| Methionine | 0.08 | Methionine[2] | 0.45 |
| Lysine (98%) | 0.08 | Cystine[2] | 0.35 |
| Threonine | 0.02 | Threonine[2] | 0.73 |
| Stone powder | 0.90 | | |
| Fish meal | 2.90 | | |
| Bran | 0.37 | | |
| Premix[1] | 1.00 | | |
| Total | 100 | | |

[1]The premix provides per kilogram of diet: VA 5,000 IU, $VD_3$ 3,300 IU, VE 62.5 mg, VK 3.6 mg, $VB_1$ 3.0 mg, $VB_2$ 9.0 mg, $VB_6$ 6.0 mg, $VB_{12}$ 0.03 mg, folic acid 60 mg, niacin 60 mg, pantothenic acid 18 mg, biotin 0.36 mg, choline chloride 600 mg, Se as $Na_2SeO_3$ 0.33mg, I as $KIO_3$ 0.35 mg, Cu as $CuSO_4 \cdot 5H_2O$ 12 mg, Mn as $MnSO_4 \cdot H_2O$ 60 mg, Fe as $FeSO_4 \cdot 7H_2O$ 80 mg, Zn as $ZnSO_4 \cdot 7H_2O$ 75 mg.

[2]Calculated values.

[3]Analysised values.

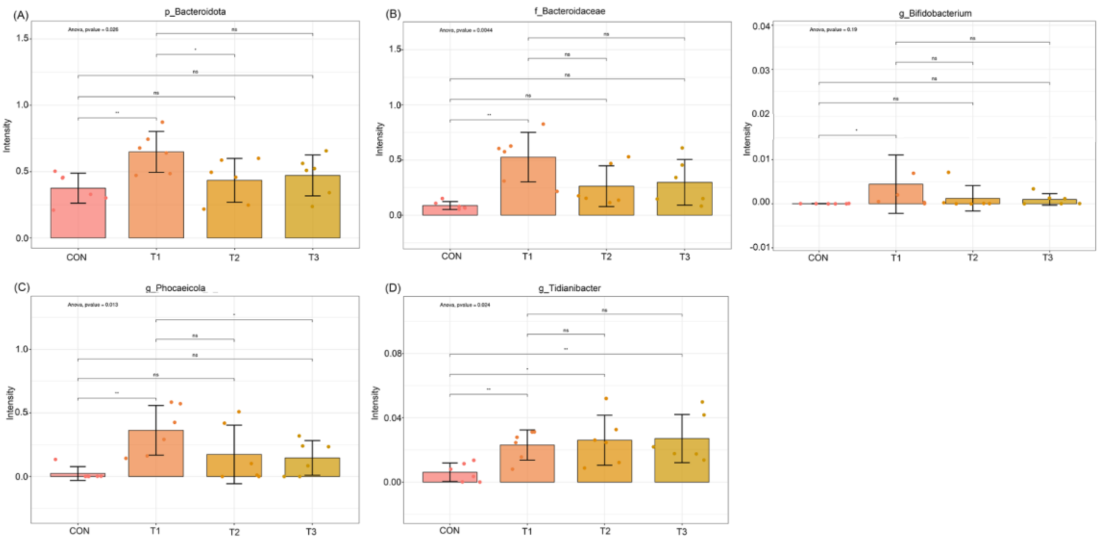


**Figure S1.** Gut Microbiota Statistical Test Bar Chart. (A) *p__Bacteroidota* Statistical test bar chart; (B) *f__Bacteroidaceae* Statistical test bar chart; (C) *g__Bifidobacterium* Statistical test bar chart; (D) *g__Phocaeicola* Statistical test bar chart; (E) *Tidjanibacter* Statistical test bar chart.

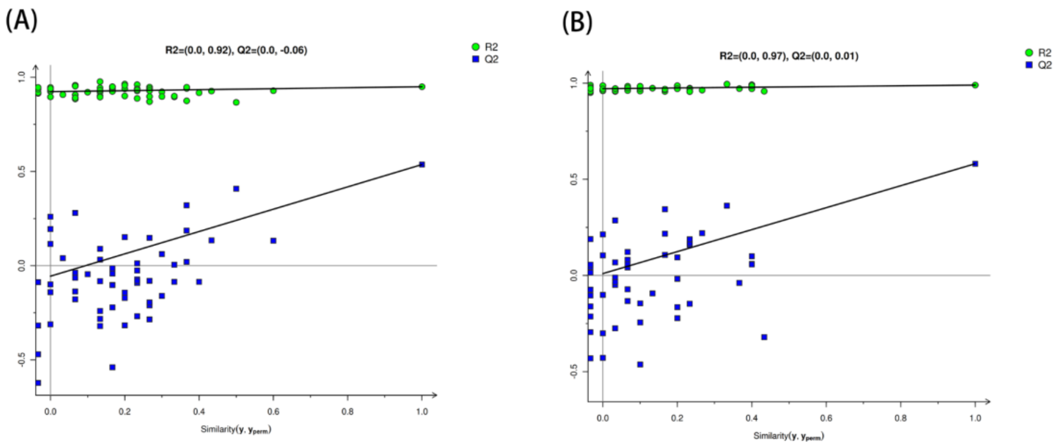


**Figure S2.** Test results of arrangement under positive and negative ion modes .(A) The arrangement test results under positive and negative ion modes; (B) The arrangement test results in negative ion mode.

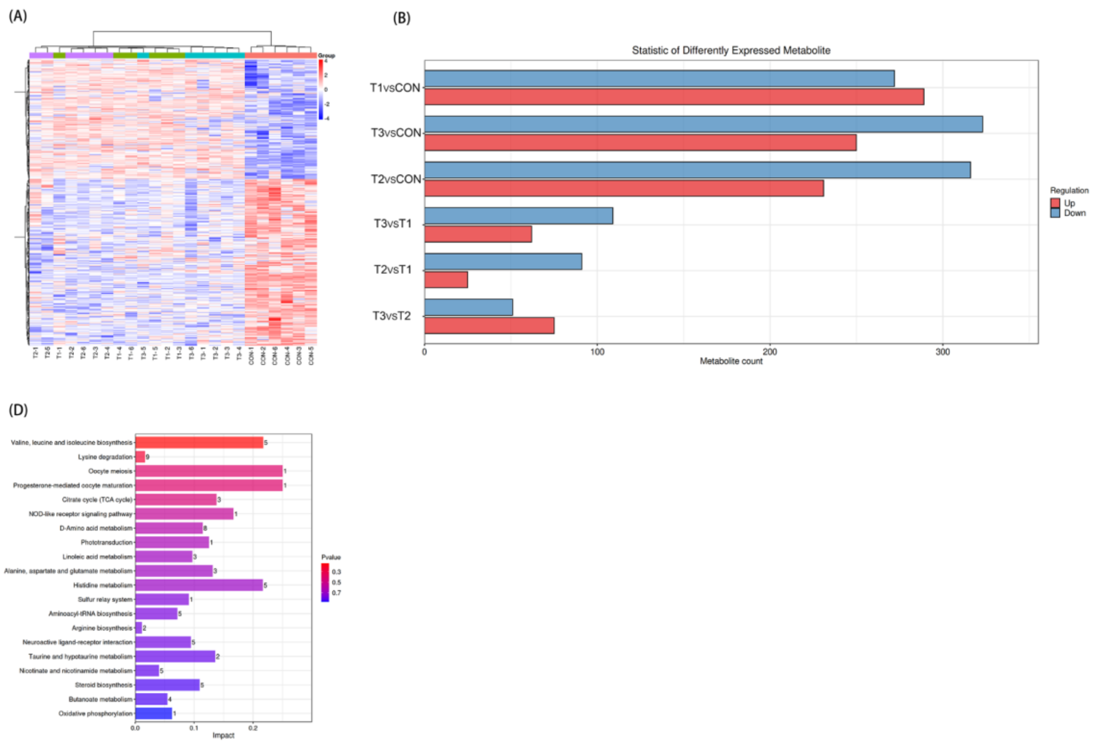


**Figure S3.** Differential metabolite overall analysis. (A)Overall metabolite clustering heat map, with gradient colors representing the magnitude of quantitative values, the redder the color, the higher the expression level, and the bluer the color, the lower the expression level. (B) The quantity of differential metabolites between groups;(C)The Venn diagram of differential metabolites, with the dots and lines below representing the selected comparison group set, the left bar chart showing the number of differential metabolites identified in the comparison group, and the right bar chart showing the number

of differential metabolites jointly identified only in the selected comparison group set.

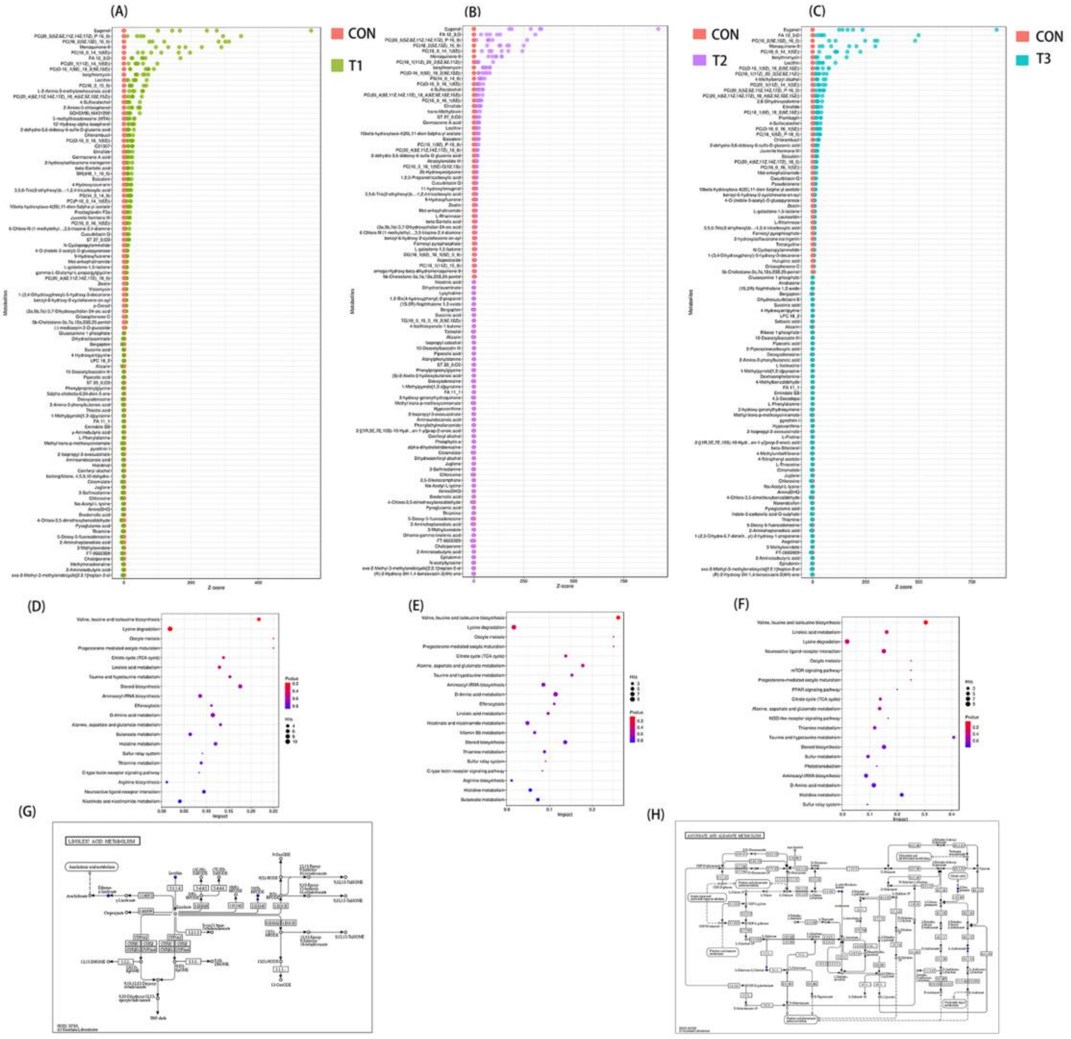


**Figure S4.** Comparative analysis of metabolites and metabolic pathways among different additive groups. (A)T2 vs. T1 Z-score figure; (B)T3 vs. T1 Z-score figure; (C)T3 vs. T2 Z-score figure; (D)T2 vs. T1 Bubble chart of metabolic pathway influencing factors; (E)T3 vs. T1 Bubble chart of metabolic pathway influencing factors; (F)T3 vs. T2 Bubble chart of metabolic pathway influencing factors;(G-H)KEGG enrichment pathway diagram, with boxes representing protein molecules, circles representing metabolic molecules, and blue representing metabolites.

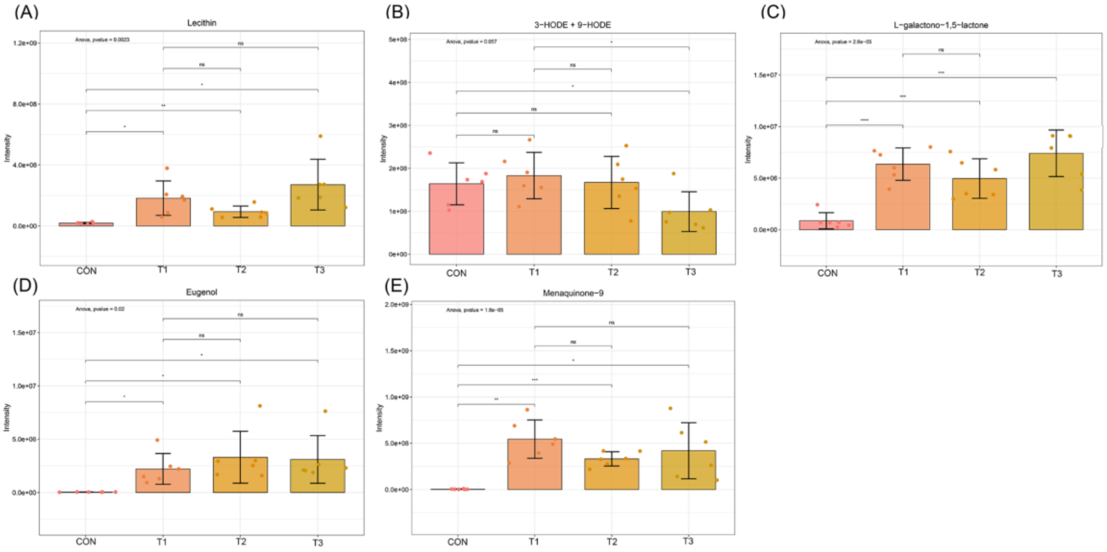


**Figure S5.** Statistical Test Bar Chart of Intestinal Metabolites. (A) Lecithin Statistical test bar chart; (B) 3-HODE+9-HODE Statistical test bar chart; (C) L-galactono-1,5-lactone Statistical test bar chart; (D) Eugenol Statistical test bar chart; (E) Menaquinone-9 Statistical test bar chart.
